# Blood orange juice intake changes specific bacteria of gut microbiota associated with cardiometabolic biomarkers

**DOI:** 10.3389/fmicb.2023.1199383

**Published:** 2023-07-04

**Authors:** Telma Angelina Faraldo Corrêa, Eric de Castro Tobaruela, Vinicius Cooper Capetini, Bruna Jardim Quintanilha, Ramon Vitor Cortez, Carla R. Taddei, Neuza Mariko Aymoto Hassimotto, Christian Hoffmann, Marcelo Macedo Rogero, Franco Maria Lajolo

**Affiliations:** ^1^Department of Food and Experimental Nutrition, School of Pharmaceutical Sciences, University of São Paulo, São Paulo, Brazil; ^2^Food Research Center (FoRC), São Paulo, Brazil; ^3^Department of Nutrition, School of Public Health, University of São Paulo, São Paulo, Brazil; ^4^Department of Clinical Analyses and Toxicology, School of Pharmaceutical Sciences, University of São Paulo, São Paulo, Brazil

**Keywords:** microbiome, short-chain fatty acids, flavanones, anthocyanins, cardiometabolic diseases

## Abstract

Blood orange juice is an important source of flavanones and anthocyanins, mainly hesperidin, narirutin, and cyanidin-3-*O*-glucoside. The benefits of these bioactive compounds have been reported, but the mechanistic details behind their biological effects are not well established. This study investigated the effects of Moro orange (*Citrus sinensis* L. Osbeck) juice (MOJ) on gut microbiota composition and cardiometabolic biomarkers in overweight women. In this study, 12 overweight women (BMI from 25.0 to 29.9 kg/m^2^), aged 18–37 years, consumed 500 mL of MOJ every day for 4 weeks. We assessed the gut microbiota composition, levels of short-chain fatty acids (SCFAs), cardiometabolic biomarkers, and insulin resistance (HOMA-IR) at baseline and after 2 weeks and 4 weeks of MOJ intake. The results suggested that MOJ intake affected the abundance of specific operational taxonomic units (OTUs) of the gut microbiota but did not significantly alter the diversity and general composition of the gut microbiota. However, MOJ intake increased the production of SCFAs, especially propionic and isobutyric acids, and significantly improved cardiometabolic biomarkers such as blood pressure and plasma VCAM-1 levels in the overweight women. Additionally, we observed significant associations between gut microbiota OTUs belonging to the Bacteroidetes phyla and *Prevotella 9* genera and the cardiometabolic biomarkers. Furthermore, MOJ reduced fasting glucose and insulin levels and HOMA-IR values, thereby enhancing insulin sensitivity in the insulin-resistant overweight women. Finally, we highlighted the importance of orange juice intake duration because some beneficial changes such as blood pressure improvements were evident at the 2-week time interval of the intervention, but other changes became significant only at the 4-week interval of MOJ intake. In conclusion, our study demonstrated that changes in specific OTUs of the gut microbiota in response to MOJ intake were associated with significant improvements in some cardiometabolic biomarkers and SCFA levels in overweight women with insulin resistance.

## 1. Introduction

Cardiovascular diseases (CVDs) and diabetes are the main causes of mortality worldwide, accounting for nearly 31% of all deaths globally. The development of CVDs is associated with risk factors such as dietary patterns, diabetes mellitus, dyslipidemia, hypertension, and obesity, whereas insulin resistance is directly associated with diabetes (World Health Organization, 2020).

Orange juice is an important source of bioactive compounds such as flavonoids, vitamin C, and carotenoids. Hesperidin and naringin are the most abundant flavanones in orange juice (Yi et al., 2017). Furthermore, blood orange (*Citrus sinensis* L. Osbeck var. Moro) juice also contains higher levels of anthocyanins, especially cyanidin-3-*O*-glucoside. These nutrients and bioactive compounds may act as antioxidants, anti-inflammatories and improve glucose and lipid metabolism and blood pressure (D’Elia et al., 2021). Animal studies have shown that orange juice reduces the risk of CVDs by improving lipid and glucose metabolism, fat storage, inflammation and hypertension (Al-Okbi et al., 2018; Berger et al., 2020; Mas-Capdevila et al., 2020; Yang et al., 2022). However, human studies are not conclusive regarding the beneficial role of orange juice in reducing the risk of CVD (Corrêa et al., 2019; Lima et al., 2019; Stevens et al., 2019; Fidélix et al., 2020; Mas-Capdevila et al., 2020).

Gut microbiota is involved in several cardiometabolic diseases (Fan and Pedersen, 2021). Disruption of gut microbiota may extract more energy from the diet, reduce satiety, promote changes in lipid and glucose metabolism, increase the production of branched-chain fatty acids (BCFAs) and trimethylamine oxide (TMAO), and increase gut permeability. The disturbance of the intestinal barrier function activates the immune system, triggering metabolic endotoxemia, low-grade inflammation, and insulin resistance (Fan and Pedersen, 2021). Gut microbiota has emerged as a modifiable therapeutic target to reduce cardiometabolic risk (Chambers et al., 2018).

Recent evidence has shown that orange flavanones could modulate the gut microbiota composition and reduce the risk of metabolic diseases (Lima et al., 2019; Fidélix et al., 2020; Nishioka et al., 2021; Santana et al., 2022). However, only one clinical trial has investigated the effects of blood orange juice on the gut microbiota composition and its effects on the host (Santana et al., 2022). In view of the above, this study evaluated the effects of daily consumption of blood orange juice (*Citrus sinensis* L. Osbeck var. Moro) on the microbiota composition and subsequent changes in the levels of cardiometabolic biomarkers in overweight women.

## 2. Materials and methods

### 2.1. Blood orange juice

The pasteurized blood orange juice (*Citrus sinensis* L. Osbeck var. Moro) was provided by Fundecitrus (Araraquara, São Paulo, Brazil) in 500 mL bottles. Moro orange juice (MOJ) was prepared as described by Brasili et al. (2017) from fruits grown in the Minas Gerais state (Brazil). Since blood oranges require low temperature for anthocyanin production, Moro orange fruits (MOJ) were stored at 9°C until the pulp reached a darker purple color, as described by Carmona et al. (2017). The detailed chemical characterization of the MOJ (quality parameters, soluble sugars, organic acids, total phenolic compounds, dietary fiber, and flavonoids) was performed according to the methods described in the Supplementary material, and the results are shown in Supplementary Tables S1, S2. The orange juice bottles were stored at −80°C until the beginning of trial.

### 2.2. Study population

This study enrolled 12 women (18–37 years) who were overweight (body mass index–BMI ranged from 25.0 to 29.9 kg/m^2^). The study subjects did not have any history of metabolic diseases and were nonsmokers, non-pregnant, non-vegetarians, and non-athletes. They also did not have any history of receiving drug therapies or dietary supplements (e.g., vitamins and minerals). The study volunteers were provided with the orange juice bottles and instructed to store their bottles in a refrigerator. The study was conducted in accordance with the Declaration of Helsinki and written informed consent was provided by volunteers. This study was approved by the Ethics Board, School of Public Health, University of São Paulo (CAAE 69382217.9.0000.5421), and was registered at the Brazilian Registry of Clinical Trials (UTN: U1111-1241-4,665).

### 2.3. Study protocol

**Figure 1:**
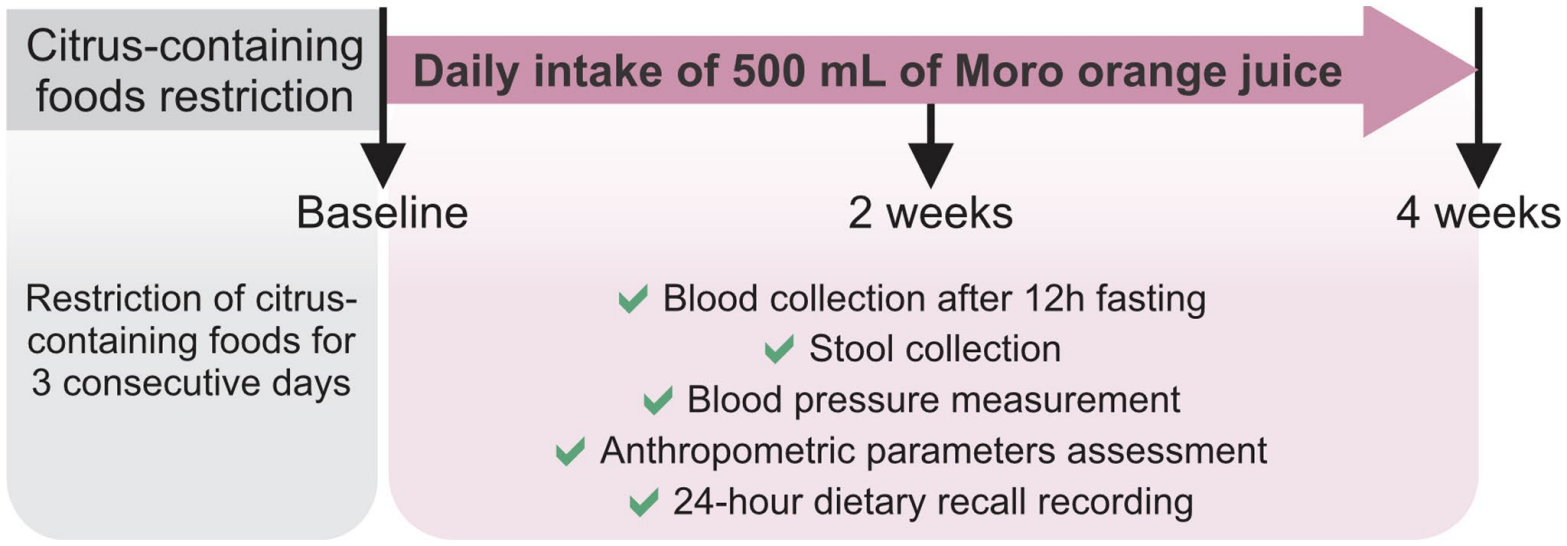
Study protocol. Twelve overweight women were selected to daily intake of 500 mL of Moro orange juice for 4 weeks. Blood and stool samples were obtained in baseline, and after 2 weeks and 4 weeks of the intervention. Blood pressure and anthropometric parameters were also measured in the same time points. Twenty-four-hour dietary recalls were recorded to food intake evaluation.

The volunteers were instructed to restrict their intake of citrus (orange, lemon, grapefruit) and their derivatives for 3 consecutive days before the 4-week longitudinal intervention study. On the first day of the intervention (baseline; A), blood samples were collected from all the volunteers after 12 h overnight fasting. A stool sample was obtained by each volunteer the day before blood collection, stored under refrigeration and delivered to the laboratory at the time of blood collection. Blood pressure (Omron HEM-7113, Kyoto, Japan) and the anthropometric parameters were also measured. The 24 h dietary recall (R24h) was the method conducted to collect diet data, to evaluate the consumption of food nutrients and to check for possible variations in food intake. Subsequently, the volunteers consumed 500 mL of pasteurized MOJ every day for 4 weeks. We further collected blood and stool samples, and assessed the blood pressure and anthropometric assessments at the 2-week (B) and 4-week (C) time points during the MOJ intake. Volunteers were instructed to maintain their usual dietary habits and lifestyle throughout the intervention period and avoid intake of any citrus-derived foods or beverages in addition to the MOJ intake as part of the study protocol. This study protocol is detailed in Figure 1.

### 2.4. Anthropometric and dietary intake measurements

Height, weight, and abdominal circumference of all the volunteers were measured at baseline (A) and at 2-week (B) and 4-week (C) times points during the MOJ intake. BMI classification was performed according to the World Health Organization (2000). The dietary intake of macronutrients and micronutrients was determined using the diet data obtained from the R24h conducted at each time point during MOJ intake and the Avanutri® 4.0 software (Rio de Janeiro, Brazil).

### 2.5. Characterization of cardiometabolic biomarkers

Plasma samples were obtained from the blood samples that were collected from the volunteers at baseline (A) and at the 2-week (B) and 4-week (C) time points of MOJ intake. Plasma samples were aliquoted and stored at −80°C until analyzes. Plasma samples were used to measure the levels of fasting glucose, insulin, total cholesterol, high-density lipoprotein (HDL), triglycerides, and inflammatory biomarkers such as C-reactive protein (CRP), interleukin 6 (IL-6), tumoral necrosis factor alpha (TNF-α), soluble vascular cellular adhesion molecule 1 (VCAM-1), soluble intercellular adhesion molecule 1 (ICAM-1), and lipopolysaccharide (LPS). Low-density lipoprotein (LDL) concentrations were calculated using the Friedewald equation (Friedewald et al., 1972). The value of 2.71 for the Homeostasis Model Assessment for Insulin Resistance (HOMA-IR) was considered as a cutoff for classifying subjects with insulin resistance and was based on a previous study on the Brazilian population (Geloneze and Tambascia, 2006).

### 2.6. Gut microbiota profiling

Fecal samples were collected by the volunteers on the day before each interview using the ColOff® (Stoll Collection Device; Zymo Research, CA, United States). The refrigerated samples were delivered to the laboratory, aliquoted in sterilized microtubes, and stored at−80°C until further analyzes.

The gut microbiota composition was evaluated by performing 16S rRNA gene sequencing analysis. Firstly, total DNA was extracted from the fecal samples using the QIAamp DNA Stool Mini kit (Qiagen, CA, United States) according to the manufacturer’s instructions. DNA quantity was estimated by measuring the absorbance of the samples at 260 nm in a NanoDrop ND-1000 spectrophotometer (Thermo Scientific, MA, United States) and using the Qubit® dsDNA HS (High Sensitivity) Assay (Life Tecnologies, CA, United States).

The 16S rRNA gene library was prepared using the 16S Metagenomic Sequencing Library Preparation Kit (Illumina, CA, United States). The V4 region of the 16S rRNA gene (25 cycles) was amplified by polymerase chain reaction (PCR) using the following primer set: 341F (5’-TCGTCGGCAGCGTCAGATGTGTATAAGAGACAG-3′) and 785R (5’-GTCTCGTGGGCTCGGAGATGTGTATAAGAGACAG-3′) (Klindworth et al., 2013). The Illumina adapter was used to build the 16S sequence library according to the protocol provided by Illumina. AccuPrime Taq DNA Polymerase System (Life Technologies) was used for the PCR. The PCR protocol for amplifying 16S rDNA was as follows: denaturation for 2 min at 94°C; 25 cycles of denaturation for 30 s at 94°C, primer annealing for 30 s at 55°C, and extension for 1 min at 68°C; final maintenance of the PCR amplified samples at 4°C. PCR was performed in a Veriti PCR Thermal cycler (Applied Biosystems, CA, United States). The PCR products were cleaned up with 10 mM Tris pH 8.5, AMPure XP beads, and 80% ethanol followed by an index PCR using the 2x KAPA HiFi HotStart ReadyMix and Nextera XT index primers (N7XX and S5XX; Nextera XT Index kit, Illumina). The clean-up was repeated again using the same reagents. The amplicons were pooled and paired-end 250 sequencing with a density of 820 k·mm^−2^ was performed on the Illumina MiSeq (Illumina, CA, USA) using the Illumina MiSeq clamshell style cartridge kit V2 for 500 cycles.

Bioinformatics analysis was performed using the Brazilian Microbiome Project (BMP) pipeline (Pylro et al., 2014) that included a combination of the VSEARCH (Rognes et al., 2016) and QIIME (Caporaso et al., 2010) software. VSEARCH software was used to remove barcodes and primer sequences from the fastq file, filter sequences by length (fastq_trunclen 400) and quality (fastq_maxee 0.5), sort by abundance, and remove singletons. Subsequently, the Operational Taxonomic Units (OTUs) were clustered and the chimeras were removed. We generated a fastq file with filtered sequences and an OTU table in.txt. Taxonomy assignments for the OTUs were performed using the uclust method in the QIIME software (version 1.9.0) and the SILVA 16S Database (version n132) was used for the reference sequences (Quast et al., 2012). The OTU table file was then converted to BIOM, and taxonomy metadata was added. The sequences were aligned and filtered, and the phylogenetic tree was constructed. QIIME software was then used to calculate alpha and beta diversity. The nucleotide sequence data was reported in the NCBI database and is available under BioProject PRJNA508648.

### 2.7. Analysis of short-chain fatty acids

The analysis of SCFAs was performed as previously described (Menezes et al., 2010) with some modifications. Frozen fecal samples (500 mg) were extracted with 1.5 mL of acetonitrile that included 0.05% 2-methyl-valeric acid (internal standard) (Sigma-Aldrich, WI, United States) and 12% HClO_4_, centrifuged at 11,000 × g for 20 min at 4°C, and filtered with the 0.2 μm PVDF filters (Millipore, MA, United States). The supernatants were injected (3 μL; split 1:10) into a Plus HP 6890 CG system (Hewlett-Packard, DE, United States) coupled to a flame ionization detector (FID) and a capillary-fused silica column (CP7747, Varian, CA, United States). The injector and FID temperatures were maintained at 270°C and 300°C, respectively. The analysis was performed using a temperature ramp from 115°C to 250°C (13 min) under constant pressure. Identification and quantification of the SCFAs was performed by comparing with a mixture of external standards (Volatile free acid mix, Supelco, PA, United States).

### 2.8. Statistical analysis

Statistical analyzes were performed using the GraphPad Prism software version 8.1.2 (GraphPad Prism software, California, United States) and the R statistical software (R Development Core Team, 2014). Normality of the data distribution was verified using the Kolmogorov–Smirnov test. The normally distributed data was analyzed using the analysis of variance (ANOVA) for repeated measurements and subsequently verified using the Tukey test. The non-normally distributed data was analyzed using the Friedman test.

The mean observed richness, Chao1, Shannon, Simpson, and Equitability values were compared between the groups using the Wilcoxon-Mann Whitney test (Fay and Proschan, 2010). A heatmap was constructed for the 20 most abundant OTUs using the Ward’s hierarchical clustering method (ward. d2) (Murtagh and Legendre, 2014). Principal Coordinates Analysis (PCoA) was used to compare the similarities between samples, and the differences were estimated using the Permutational analysis of variance (PERMANOVA) (Anderson, 2001). The analyzes were performed with the R statistical software (R Development Core Team, 2014) using the qiimer, ggplot2 (Wickham, 2016), phyloseq (McMurdie and Holmes, 2013), and vegan (Oksanen et al., 2019) packages.

Spearman correlation analysis was performed to evaluate the association between changes in the cardiometabolic biomarkers and changes in the specific groups of bacteria in response to MOJ intake. *p* < 0.05 was considered as statistically significant.

## 3. Results

### 3.1. Characterization of Moro orange juice composition

Twelve overweight women (BMI 27.86 ± 0.41 kg/m^2^) consumed 500 mL/day of blood orange juice (*Citrus sinensis* L. Osbeck var. Moro) for 4 weeks. The chemical composition of MOJ is presented in Supplementary Tables S1–S3. The concentrations of sucrose (14.00 ± 0.72 mg/500 mL) and citric acid (5.81 ± 0.23 g/500 mL) were highest among the soluble sugars (31.95 mg/500 mL) and organic acids (7.47 g/500 mL), respectively. The total dietary fiber content was 1.70 g/500 mL, including 0.40 ± 0.03 g/500 mL of soluble dietary fiber and 1.30 ± 0.06 g/500 mL of insoluble dietary fiber. The total concentration of flavonoids in the MOJ was 290.95 mg/500 mL including high concentrations of hesperidin (182.25 mg/500 mL) and narirutin (20.95 mg/500 mL). Cyanidin-3-*O*-glucoside (61.15 ± 8.80 mg/500 mL) as the main anthocyanin in the MOJ. Overall, our analysis showed that each study volunteer consumed 215.35 mg of flavanones and 75.60 mg of anthocyanins per day.

### 3.2. Moro orange juice intake alters specific OTUs of the gut microbiota composition

The relative abundance of OTUs was analyzed to evaluate the effects of the daily intake of MOJ on the gut microbiota profile. We obtained 5,735,751 sequences and 12,413 clustered OTUs with 97% similarity from the fecal samples of 12 overweight women after quality control and bioinformatics analysis. The number of sequences varied significantly between the samples and ranged from 42,445 to 269,320 sequences per sample. Therefore, all the samples were rarefied to 38,000 reads and were subjected to alpha and beta-diversity analysis.

**Figure 2:**
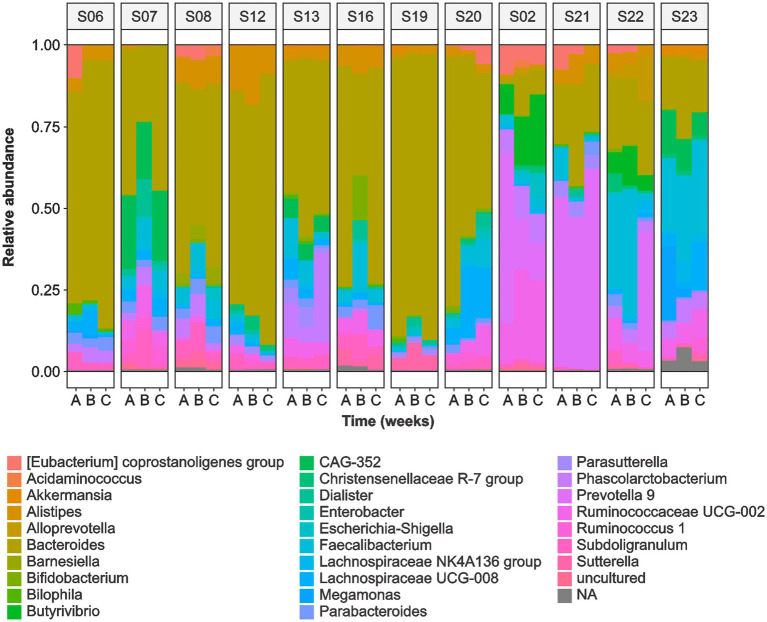
Relative abundance of the top 60 gut microbiota genera in the fecal samples of 12 overweight women at baseline **(A)**, at 2-weeks **(B)** and 4 weeks **(C)** of daily blood orange juice intake.

**Figure 3:**
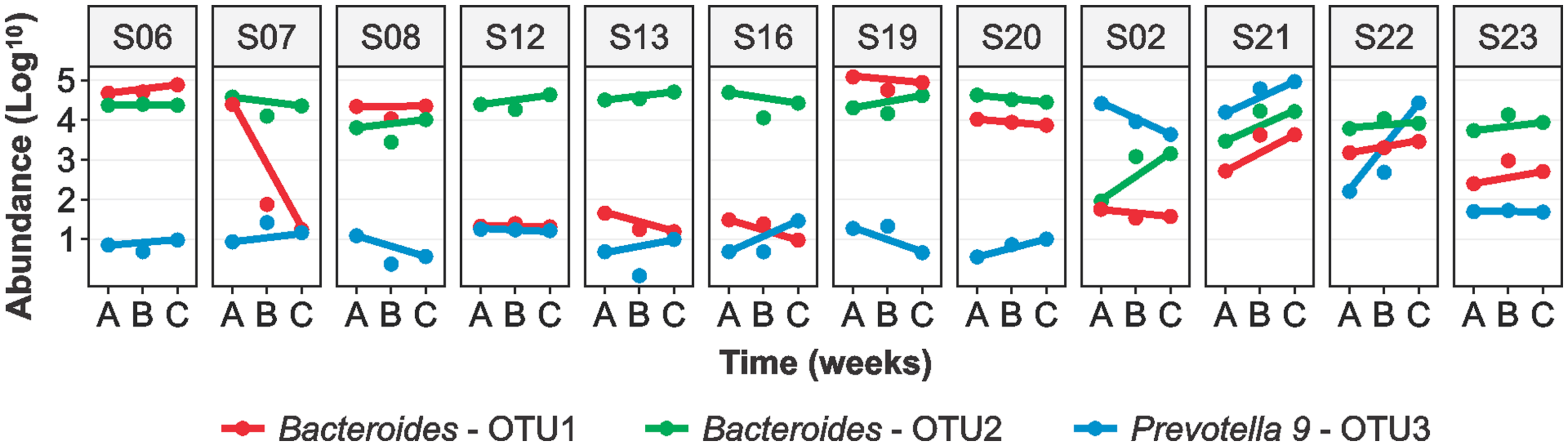
Logarithmic (log10) abundance of the top 3 OTUs in fecal samples of the 12 overweight women at baseline **(A)**, at 2-weeks **(B)** and 4-weeks **(C)** of daily blood orange juice intake. OTU: Operational Taxonomic Unit.

The sequence analysis identified the following phyla: Bacteroidetes (52.5%), Firmicutes (40.6%), Proteobacteria (3.5%), Actinobacteria (0.7%), and Verrucomicrobia (0.4%). *Bacteroides* (37.9%) was the most abundant genera followed by *Prevotella 9* (5.3%), *Faecalibacterium* (4.9%), *Alistipes* (3.0%), *Ruminococcaceae UCG-002* (2.8%), and *Phascolarctobacterium* (2.6%) (Figure 2). We observed significant variability in the microbial communities between the volunteers (Supplementary Figure S1). Therefore, we selected three most abundant OTUs and compared their microbiota profiles throughout the intervention. Two of the three OTUs belonged to *Bacteroides* (OTU1 and OTU2) whereas OTU3 belonged to the *Prevotella 9* (Figure 3). These three OTUs represented 21.4% (OTU1: 8%; OTU2: 10.1%; OTU3: 3.3%) of the total microbial communities. In summary, gut microbiota profiles of 9 volunteers were similar, and showed high relative abundance of OTU1, low abundance of OTU3, and variable abundance of OTU2. The gut microbiota profiles of the remaining three volunteers (S02, S21, and S22) showed variations from this pattern. The gut microbiota profile of volunteer S22 showed similar pattern at baseline but higher OTU3 abundance after 4 weeks of MOJ intake, whereas the gut microbiota profiles of the remaining 2 volunteers (S02 and S21) showed higher abundance of OTU3 and lower abundance of OTU1 throughout the intervention.

Alpha-diversity analysis showed that the number of gut microbial species ranged from 312 to 2,752 (Supplementary Table S6). Chao1 index ranged from 418 to 4,005 species for the study samples; Shannon index of species diversity ranged from 3.54 to 8.17; and the Simpson diversity index ranged from 0.77 to 0.99. We did not observe significant differences in alpha-diversity between the gut microbiota profiles at baseline (A) and at 2-weeks (B) and 4-weeks (C) after MOJ intake (Supplementary Table S6). Beta-diversity also showed high stability of the gut microbiota composition in each volunteer throughout the intervention. This suggested that the 4-week timeline of MOJ intake (500 mL) may not have been sufficient to assess changes in the gut microbiota diversity, based on the metrics used in this study which are shown in Supplementary Figures S2, S3.

Procrustes analysis was performed using weighted UniFrac distance matrix and PCoA ordination and the gut microbiota communities was clustered into two groups during all the time points of the experiment. Procrustes analysis between ordinations from samples A (baseline) and B (2 weeks), and samples B and C (4 weeks) showed significant differences (*p* = 0.001 and 0.001, respectively). The samples with high abundance of *Bacteroides* and *Prevotella* were clustered in Group 1 (red) and Group 2 (blue), respectively. The samples clustered in Group 1 tended to remain within the same group at both A and B time points (Supplementary Figure 3A). However, samples in Group 2 showed variations at different time points. Volunteers 2 and 21 remained in Group 2, whereas volunteers S22 and S23 changed from Group 2 to Group 1 (red arrows). Similar results were observed between time points B to C. However, volunteer S22 changed from Group 1 to Group 2 between time points B to C (Supplementary Figure 3B).

### 3.3. Moro orange juice intake significantly alters metabolism of the gut microbiota

The intake of MOJ did not significantly alter the gut microbiota composition in the study volunteers but affected metabolism of the gut microbiota as evidenced by the changes in the concentrations of SCFAs in the fecal samples. The levels of SCFAs including acetic acid, propionic acid, butyric acid, isobutyric acid, isovaleric acid, valeric acid, and hexanoic acid in the fecal samples at baseline and at 2-weeks and 4-weeks after MOJ intake is shown in Table 1. Acetic acid, propionic acid, and butyric acid were the most abundant SCFAs (74.5%) in the fecal samples. MOJ intake increased the levels of all SCFAs except for hexanoic acid. The concentration of acetic acid in the fecal samples increased at 2 weeks compared to the baseline but was restored to baseline levels at 4-weeks after intake of MOJ. The levels of propanoic acid and isobutyric acid were significantly higher in the 4-week time interval of the MOJ intake compared to the baseline (*p* < 0.05). The levels of other SCFAs also increased after MOJ intake but did not show statistically significant differences when compared with the baseline concentrations. Furthermore, the total SCFAs levels were elevated in the fecal samples after MOJ intake but did not show statistically significant differences when compared with the baseline concentrations.

**Table 1:** Effect of blood orange juice intake on the levels of short-chain fatty acids in the fecal samples.

Fecal SCFAs (μmol/g)	Baseline	2 weeks	4 weeks	Value of *p*
Acetic acid	11.01 ± 1.22	12.25 ± 0.94	11.51 ± 0.85	0.338
Propionic acid	**5.23 ± 0.47** ^**b**^	**6.55 ± 0.81** ^**ab**^	**6.60 ± 0.52** ^**a**^	**0.028**
Butyric acid	7.01 ± 1.18	9.67 ± 1.71	9.33 ± 1.38	0.264
Isobutyric acid	**1.75 ± 0.15** ^**b**^	**2.26 ± 0.27** ^**ab**^	**2.46 ± 0.27** ^**a**^	**0.019**
Valeric acid	2.08 ± 0.15	2.35 ± 0.18	2.43 ± 0.19	0.076
Isovaleric acid	2.42 ± 0.26	3.15 ± 0.44	3.03 ± 0.43	0.264
Hexanoic acid	1.80 ± 0.20	1.66 ± 0.16	1.71 ± 0.17	0.517
Total SCFAs	31.29 ± 2.88	37.90 ± 3.40	37.07 ± 3.04	0.205

Results are expressed as the mean ± standard error. The value of *p*s were calculated using the Friedman test. Values in bold and different superscript letters indicate statistical significance (*p* < 0.05) among values obtained in the baseline and after 2 and 4 weeks of blood orange juice intake.

### 3.4. Moro orange juice intake alters anthropometric parameters and levels of few cardiometabolic biomarkers

Recent studies reported that changes in the gut microbiota composition are associated with metabolic changes in the human body including body composition parameters and cardiometabolic biomarkers in response to specific changes in the gut microbiota (Lima et al., 2019; Fidélix et al., 2020; Kahleova et al., 2020; Asnicar et al., 2021).

In this study, we analyzed anthropometric parameters, cardiometabolic biomarkers, and consumption of food nutrients (R24h) at baseline and at 2-weeks and 4-weeks of MOJ intake. The daily intake of energy, macronutrients, and micronutrients were comparable between the baseline and the intervention time-points (*p* > 0.05), but we observed changes in the intake of dietary fiber, vitamin C, and potassium (Supplementary Table S7). The dietary fiber levels were significantly reduced at 4 weeks of MOJ intake compared to the baseline (20.26 ± 2.70 to 13.51 ± 1.52 g/100 g; *p* = 0.039), whereas we observed significant increase in the levels of vitamin C (66.55 ± 27.95 to 278.13 ± 18.55 mg/100 g; *p* < 0.001) and potassium (2.26 ± 0.23 to 2.82 ± 0.11 g/100 g; *p* = 0.039) at 2 weeks of MOJ intake compared to the baseline.

The effects of MOJ intake for 4 weeks on the anthropometric variables and the cardiometabolic biomarkers are shown in Table 2.

**Table 2:** Anthropometric, biochemical, and cardiometabolic biomarkers of overweight women at baseline and at 2-week and 4-week time points during the intake of blood orange juice.

Variables	Baseline	2 weeks	4 weeks	Value of *p*
Body weight (kg)	73.65 ± 6.36	73.88 ± 6.37	73.99 ± 6.69	0.722
BMI (kg/m^2^)	27.86 ± 0.41	27.95 ± 0.45	27.99 ± 0.52	0.938
Abdominal circumference (cm)	90.00 ± 1.55	88.75 ± 1.50	88.92 ± 1.51	0.741
Blood pressure (mmHg)				
Systolic	118.67 ± 2.55	113.92 ± 3.93	113.83 ± 4.42	0.338
Diastolic	**73.08 ± 2.35** ^**a**^	**69.25 ± 2.77** ^**b**^	**69.25 ± 1.89** ^**b**^	**0.020**
Glucose (mg/dL)	79.58 ± 3.36	75.08 ± 2.40	74.75 ± 3.36	0.537
Insulin (μUI/mL)	17.05 ± 1.96	15.25 ± 1.37	14.21 ± 1.22	0.558
HOMA-IR	3.43 ± 0.52	2.83 ± 0.27	2.56 ± 0.17	0.338
Cholesterol (mg/dL)				
HDL	50.08 ± 4.39	47.75 ± 3.42	49.83 ± 4.44	0.112
LDL	70.83 ± 6.97	76.17 ± 8.03	78.50 ± 6.36	0.338
Total	138.42 ± 9.32	144.08 ± 9.77	150.50 ± 9.02	0.717
Triglycerides (mg/dL)	88.00 ± 10.91	100.67 ± 13.67	104.75 ± 15.31	0.205
C-reactive protein (mg/dL)	2.23 ± 0.38	2.84 ± 0.57	2.20 ± 0.49	0.405
TNF-α (pg/mL)	3.10 ± 0.12	3.12 ± 0.13	3.10 ± 0.12	0.723
IL-6 (pg/mL)	3.95 ± 0.67	3.96 ± 0.72	4.15 ± 0.76	0.236
IL-10 (pg/mL)	3.83 ± 0.19	3.88 ± 0.23	3.92 ± 0.18	0.976
VCAM-1 (ng/mL)	**554.99 ± 15.63** ^**a**^	**553.62 ± 14.31** ^**ab**^	**545.31 ± 14.65** ^**b**^	**0.039**
ICAM-1 (ng/mL)	367.57 ± 25.73	353.73 ± 28.89	349.34 ± 27.69	0.920
Fibrinogen (mg/dL)	301.17 ± 23.91	273.33 ± 21.25	297.75 ± 26.81	0.205
LPS (EU/mL)	0.07 ± 0.00	0.08 ± 0.01	0.07 ± 0.00	0.875

BMI, body mass index; HOMA-IR, Homeostasis model assessment for insulin resistance; HDL, high-density lipoprotein; LDL, low-density lipoprotein; TNF-α, tumor necrosis factor-alpha; IL, interleukin; VCAM-1, vascular cellular adhesion molecule 1; ICAM-1, intercellular adhesion molecule 1; LPS, lipopolysaccharide. The *p*-values were calculated using the Friedman test. Values in bold and different superscript letters indicate statistical significance (*p* < 0.05) among values (*n* = 12) obtained in the baseline and after 2 and 4 weeks of blood orange juice intake.

We observed significant reduction (*p* = 0.02) in the diastolic blood pressure (DBP) after 2 weeks of MOJ intake (73.08 ± 2.35 to 69.25 ± 2.77 mmHg) and this effect was maintained at the 4-week time point. We also observed reduced systolic blood pressure (SBP) at 2-weeks and 4-weeks of MOJ intake compared to the baseline (118.67 ± 2.55 to 113.83 ± 4.42 mmHg), but the differences were not statistically significant. MOJ intake also reduced the levels of biomarkers of the glucose and lipid metabolism, but the differences were not significant because of large variations in the values between the study volunteers. The intake of MOJ reduced the levels of plasma glucose and insulin from 79.58 ± 3.36 to 74.75 ± 3.36 mg/dL and from 17.05 ± 1.96 to 14.21 ± 1.22 μUI/mL, respectively (*p* > 0.05). HOMA-IR values also reduced from 3.43 ± 0.52 at baseline to 2.56 ± 0.17 at the 4-week MOJ intake time points (*p* > 0.05).

**Figure 4:**
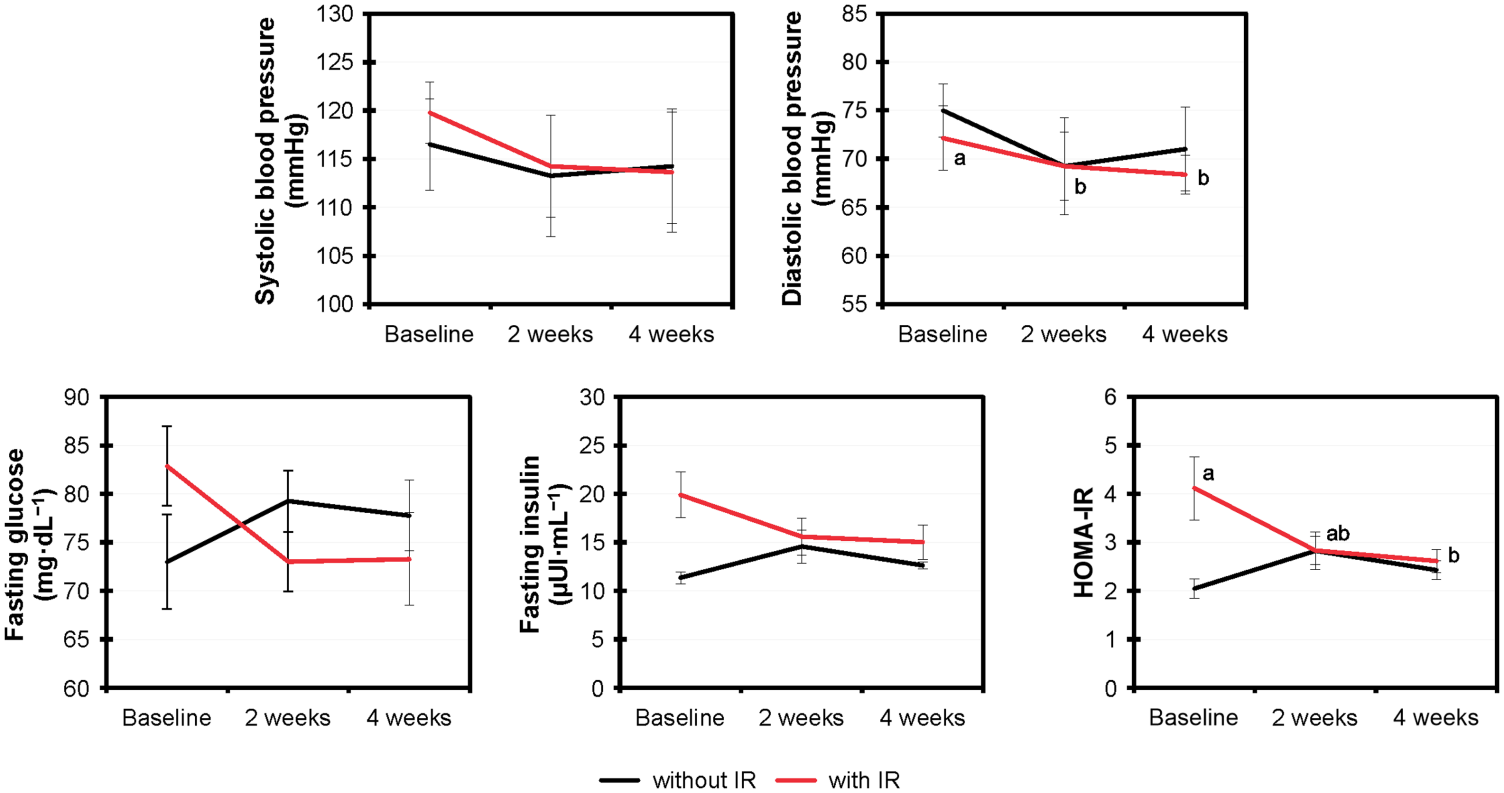
Comparative variation of the systolic and diastolic blood pressure, fasting glucose and fasting insulin levels, and HOMA-IR values in the overweight women with (*n* = 8) and without (*n* = 4) insulin resistance that drank Moro orange juice (500 mL per day) for 4 weeks. Different superscript letters indicate statistical significance calculated using the Friedman test (*p* < 0.05) by comparing the experimental values (*n* = 12) at baseline and those at 2-weeks and 4-weeks of Moro orange juice intake. HOMA-IR, Homeostasis model assessment for insulin resistance.

Based on the HOMA-IR values, 8 out of 12 volunteers showed insulin resistance and 4 showed normal insulin sensitivity. We assessed the effects of MOJ intake on the insulin resistance by comparing the fasting glucose, fasting insulin and HOMA-IR values in the volunteers with or without insulin resistance (Figure 4). Furthermore, we analyzed the effects of the MOJ intake on the SBP and DBP values in the normal and the insulin-resistant women. The intake of MOJ reduced the HOMA-IR (from 4.12 ± 0.65 to 2.62 ± 0.24, *p* = 0.012) and DBP (from 72.13 ± 3.32 to 68.38 ± 2.01 mmHg, *p* = 0.026) in women with insulin resistance. Women without insulin resistance did not show any significant changes in the cardiometabolic biomarkers. Furthermore, in the women with insulin resistance, intake of MOJ for 4 weeks reduced the plasma VCAM-1 levels (554.99 ± 15.63 to 545.31 ± 14.65 ng·mL^−1^, *p* = 0.039) but the plasma levels of ICAM-1, C-reactive protein, TNF-α, IL-6, and IL-10 were not significantly altered.

### 3.5. Cardiometabolic biomarkers show significant correlation with specific gut microbiota OTUs

**Figure 5:**
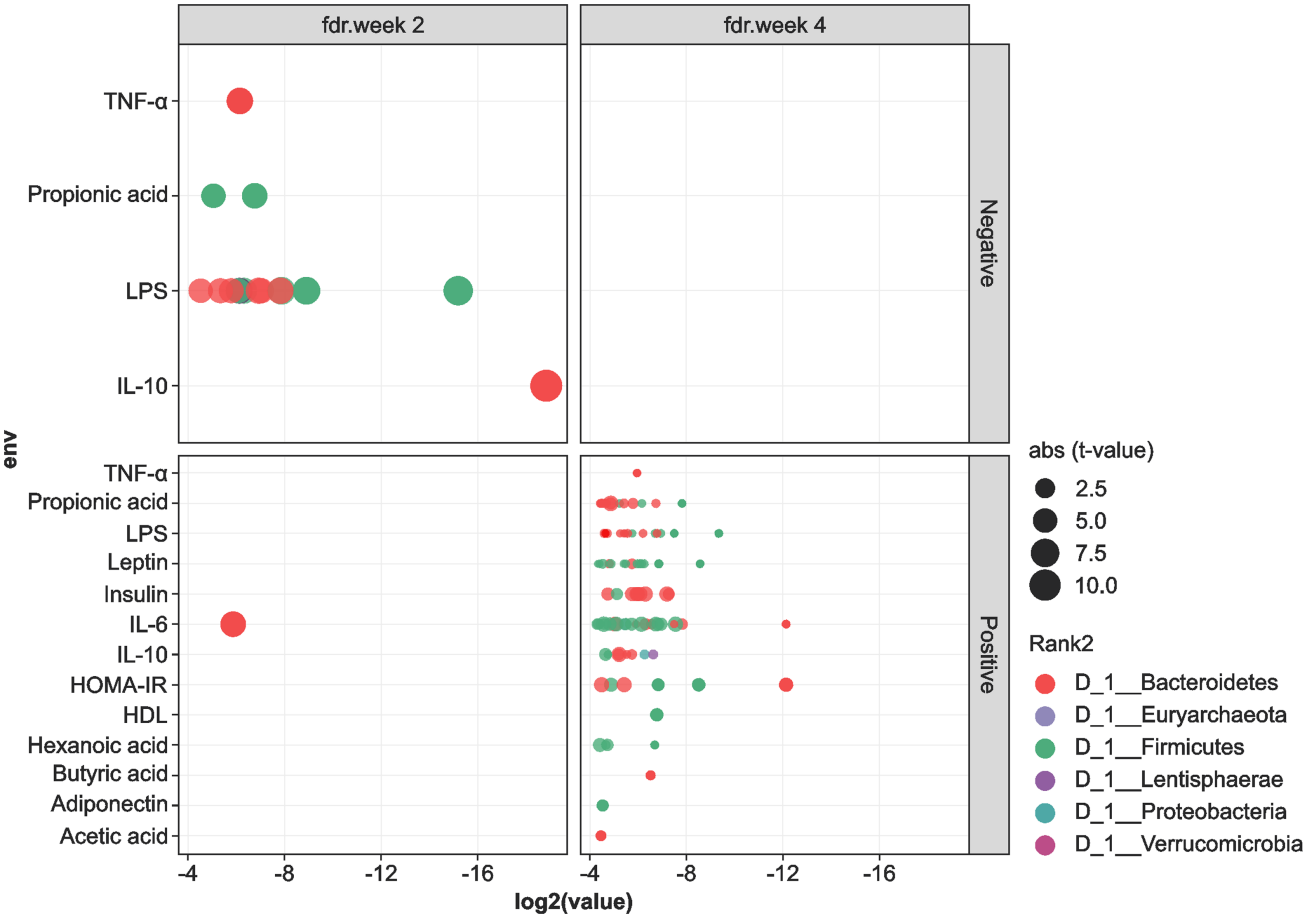
Correlation between cardiometabolic parameters and the 6 most abundant phyla in the fecal samples of the 12 overweight women volunteers that drank Moro orange juice (500 mL per day) for 4 weeks. BMI, body mass index; HOMA-IR, Homeostasis model assessment for insulin resistance; HDL, high-density lipoprotein; LDL, low-density lipoprotein; CRP, C-reactive protein; TNF-α, tumor necrosis factor alpha; IL, interleukin; VCAM-1, vascular cellular adhesion molecule 1; ICAM-1, intercellular adhesion molecule 1; LPS, lipopolysaccharide.

**Figure 6:**
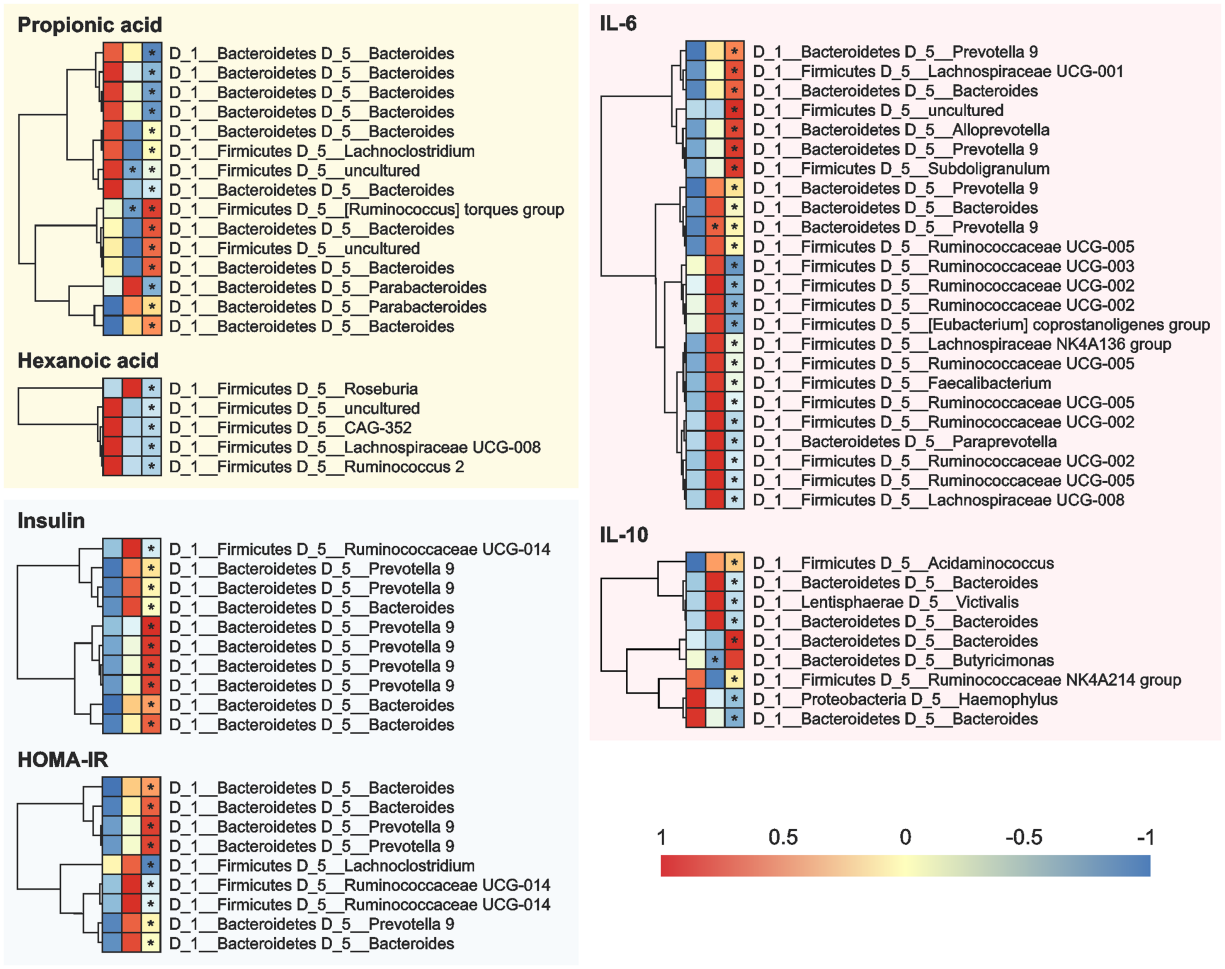
Correlation between the cardiometabolic parameters and the OTUs in the fecal samples of the 12 overweight women volunteers that drank Moro orange juice (500 mL per day) for 4 weeks. A = Baseline, B = 2 weeks, and C = 4 weeks. OUT, Operational Taxonomic Unit; HOMA-IR, Homeostasis model assessment for insulin resistance; IL, interleukin.

We performed Spearman’s correlation analysis to determine the association between the levels of cardiometabolic biomarkers and the gut microbiota OTUs, and the results are shown in Figures 5, 6. In general, OTUs classified within the Firmicutes and Bacteroidetes phyla showed negative correlations with the levels of IL-10, TNF-α, LPS, and propionic acid at the 2-week time-point during MOJ intake. Furthermore, at this time-point, only one OTU in the Firmicutes showed positive correlation with the IL-6 levels. In contrast, correlational analysis showed only positive associations between OTUs in the Firmicutes and Bacteroidetes and levels of the cardiometabolic biomarkers. Furthermore, Proteobacteria and Lentisphaerae showed positive correlations with the IL-10 levels at the 4-week time-point during MOJ intake (Figure 5).

At the genera level (Figure 6), *Bacteroides* showed positive correlations and *Prevotella 9* showed negative correlations with the levels of SCFAs (propionic and hexanoic acids), insulin, HOMA-IR, IL-6, and IL-10 at the 2-and 4-week time-points during MOJ intake. We also observed correlations between other bacterial genera and specific biomarkers measured in this study. The genus *Lachnoclostridium* showed positive correlation (*p* < 0.05) with the levels of propionic acid and negative correlation with HOMA-IR at the 4-week time-point of MOJ intake. Furthermore, the genus *Ruminococcaceae UCG-014* showed significant negative correlation with insulin levels and HOMA-IR at the 4-week time-point of MOJ intake, whereas *Ruminococcaceae UCG-002*, *Ruminococcaceae UCG-003*, and *Ruminococcaceae UCG-005* showed negative correlation with the plasma IL-6 levels.

## 4. Discussion

Blood orange juice is an important source of anthocyanins and flavanones such as hesperidin and naringin. The benefits of these bioactive compounds are well known but the mechanisms of action underlying their biological effects are not well established. The bioavailability and beneficial effects of orange juice flavonoids are significantly influenced by their chemical structure, food matrix effects, and composition of the host gut microbiota. Hesperidin and naringin are resistant to enzymatic breakdown in the stomach and the small intestine (Mas-Capdevila et al., 2020). In the colon, these flavanones are broken down by the α-rhamnosidases and β-glucosidases secreted by the gut microbiota and are converted into aglycones, hesperetin and naringenin. Aglycones are then absorbed through the intestinal epithelium or further metabolized into phenolic acids by bacterial enzymes (Mas-Capdevila et al., 2020). The two-way interaction between the flavonoids and the gut microbiota improves metabolism and reduces the risk of cardiometabolic diseases. Gut microbiota enhance the bioavailability and bioactivity of the dietary flavonoids through enzymatic metabolism. On the other hand, flavonoids act as prebiotics altering the gut microbiota composition and activity. Flavonoids suppress the growth of pathobionts and promote the growth of commensals (Corrêa et al., 2019). Recent studies have shown that flavonoids from orange juice alter the composition and activity of the gut microbiota (Stevens et al., 2019; Fidélix et al., 2020; Mas-Capdevila et al., 2020; Santana et al., 2022). The present study aimed to evaluate the effects of MOJ on the gut microbiota composition and the cardiometabolic biomarkers in overweight women.

Several recent studies have investigated the benefits of blood orange juice intake, especially of the Moro variety (Dorna et al., 2021; Nishioka et al., 2021; Ribeiro et al., 2021; Briskey et al., 2022; Santana et al., 2022; Capetini et al., 2023). However, the effects of MOJ on the gut microbiota profiles have not been investigated in detail. Nishioka et al. (2021) performed a clinical trial with MOJ by analyzing the gut microbiota composition, but stratified the volunteers based on the fecal profile of the flavanones. Santana et al. (2022) performed a randomized crossover study with obese patients that consumed ‘Pera’ orange juice (POJ) or MOJ and reported improved gut microbiota profile (initially with obesity-associated alterations) after 15 days. The improvements were significantly higher for patients who consumed MOJ than those who consumed POJ, especially in patients with higher obesity classes. The authors reported significant effects of MOJ on the gut microbiota and on several oxidative stress and inflammatory response biomarkers but did not clearly define the association between bacterial OTUs and the biomarker levels. This is the first study to report the correlation between the gut microbiota composition and the cardiometabolic biomarkers.

Gut microbiota belonged predominantly to the Bacteroidetes and Firmicutes phyla, with the *Bacteroides* genera being the most prevalent. The Firmicutes/Bacteroidetes (F/B) ratio did not change significantly throughout the study. A higher F/B ratio are positive correlated with the development of obesity and insulin resistance in overweight subjects (Liu et al., 2021). A similar profile was reported in our previous studies (Nishioka et al., 2021; Santana et al., 2022). However, these studies did not identify *Prevotella 9* as the predominant OTU. We did not classify the bacterial OTUs at the species level. However, previous studies showed that *Bacteroides dorei* and *B. vulgatus*, which belong to the phylum Bacteroidetes, are depleted in subjects with obesity. These species play an important role in maintaining a healthy gut ecosystem, protecting role against atherosclerosis, and reducing LPS activity (Yoshida et al., 2021). Yoshida et al. (2021) also showed that these species promote the catabolism of branched-chain amino acids (BCAAs), protecting against obesity. Moreover, some species of *Bacteroides* including *B. ovatus*, *B. fragilis*, *B. distasonis*, and *B. uniformis* are involved in polyphenol metabolism (*O*-deglycosylation) (Braune and Blaut, 2016; Cortés-Martín et al., 2020). *O*-deglycosylation by human gut bacteria is attributed to the activity of a specific *Parabacteroides* species, namely *P. distasonis*. Two *Parabacteroides* OTUs in the fecal samples showed significant correlation with the levels of propionic acid (Al-Ishaq et al., 2021). In contrast, none of the known *Prevotella* species have been reported to play a role in flavonoid metabolism. However, the abundance of *Prevotella* is strongly associated with plant-based diets, rich in fiber, and *Prevotella copri* is considered an important biomarker for diet (Precup and Vodnar, 2019).

Subjects with obesity generally have lower gut microbiota diversity and richness than the lean subjects (Liu et al., 2021). Low microbiota richness is associated with dyslipidemia, fat storage, insulin resistance, and inflammation (Lin et al., 2023). As well as in obesity, in prediabetes, gut microbiota composition is altered and exhibits reduced bacterial richness and reduced bacterial butyrate producers and increased species with a pro-inflammatory potential (Fan and Pedersen, 2021). Significant changes in gut microbiota composition were not observed in overweight women after 4 weeks of MOJ intake. However, significant correlations were identified between specific OTUs and cardiometabolic biomarkers, thereby highlighting the role of the gut microbiota on flavanone metabolism, and the health benefits of MOJ. MOJ intake did not significantly alter the gut microbiota diversity but increased the production of SCFAs, especially propionic acid and isobutyric acid, and induced significant improvements in several cardiometabolic biomarkers such as DBP and VCAM-1. Furthermore, *Bacteroides* and *Prevotella 9* and other specific OTUs showed positive and negative correlations with several biomarkers including SCFAs, insulin, HOMA-IR, IL-6, and IL-10. SCFAs modulate CVD risk factors by regulating appetite and improving systolic and diastolic pressure, glucose and lipid homeostasis, adiposity and inflammation. SCFAs maintain epithelium integrity and restore gut barrier function preventing the translocation of LPS. Furthermore, SCFAs induce the production of mucin which creates a physical barrier between luminal bacteria and epithelial cells (Chambers et al., 2018). SCFAs bind to G-protein-coupled receptor (GPR) 41 and GPR 43 enhancing the release of anorexigenic peptides glucagon-like peptide-1 (GLP-1) and peptide YY (PYY) from enteroendocrine cells. GLP-1 and PYY contribute to satiety, reduction of plasma glucose and lipids, insulin resistance and inflammation, and increased lipid oxidation (Van Hul and Cani, 2023). A differential association of OTUs classified under the same bacterial genus with the same biomarkers was observed. This highlighted complexity of the gut microbiota response to the orange juice intake. Moreover, although some associations were evident within 2 weeks of MOJ intake, they became significant only at the end of the intervention (4 weeks). This suggested that the changes in the OTUs in response to the constituents of the MOJ were not instantaneous but occurred gradually over a longer period of time.

MOJ intake significantly reduced DBP in the overweight women. This was consistent with the previous findings in eutrophic and overweight adults (Fraga et al., 2021) and obese adults (Santana et al., 2022; Santos et al., 2022) after Pera orange juice and MOJ intake. The relative risk of all the major CVD is significantly reduced by a reduction of 5 mmHg in DBP (Zanchetti, 2016). It is plausible that hesperidin exerts antihypertensive effects through the vascular nitric oxide (NO) synthase pathway. A previous study reported that hesperidin and its metabolites enhance NO bioavailability and protect the endothelial function by reducing the levels of the reactive oxygen species (Mas-Capdevila et al., 2020). DBP improvement may also be associated with the metabolic products of citrus flavanones such as flavanones-phase II conjugates and the phenolic acids, which are generated by the gut bacteria (Fraga et al., 2021). The flavanone composition of MOJ is comparable to that of POJ. Therefore, the health benefits of MOJ intake in the overweight women volunteers in this study may be attributed to the hesperidin and naringin metabolites.

The reduced levels of plasma VCAM-1 after MOJ intake suggested that the bioactive compounds from the MOJ reduced cardiometabolic risk by targeting the endothelium. Anthocyanins in MOJ (75.60 mg/500 mL) are metabolized by the gut microbiota and their metabolites are potentially responsible for the beneficial effects on the endothelium (Hidalgo et al., 2012; Kuntz et al., 2015; Krga and Milenkovic, 2019). A recent systematic review and meta-analysis reported that hesperidin significantly reduced the levels of VCAM-1 but did not alter the circulating E-selectin, IL-6, and ICAM-1 levels (Lorzadeh et al., 2019) similar to our findings.

The reduction in the cardiometabolic biomarkers was significantly higher in the insulin resistant women, similarly to the findings of Santana et al. (2022). This suggested significantly higher health benefits of regular MOJ intake for individuals with insulin resistance. The comparison of the effects of MOJ intake in women with or without insulin resistance showed the beneficial effects of MOJ on the cardiometabolic disorder including reduced levels of glucose, insulin, and HOMA-IR, as well as greater reduction of SBP and DBP in the insulin-resistant women. As observed in the correlation analyzes, some changes (as in blood pressure parameters) were significant at the 2-week time-point of the MOJ intake, whereas other parameters were significant only after 4 weeks. This highlighted the importance of the intake duration. Silveira et al. (2015) reported similar results in eutrophic and overweight volunteers who consumed 750 mL daily of blood orange juice for 8 weeks. The blood orange juice intake enhanced insulin sensitivity and reduced total cholesterol and LDL levels as well as SBP and DBP.

Gut microbiota is involved in the regulation of energy homeostasis, control of body weight, and inflammation. The changed microbiota composition in overweight subjects extracts more energy from the diet and increases lipogenesis. This microbiota provides lipogenic substrates to the liver and increases the lipoprotein lipase activity, as a consequence of the Fasting-Induced Adipose Factor (FIAF) suppression in the gut. Consequently, fatty acids and triacylglycerol are released from circulating lipoprotein in muscle, and adipose tissue, increasing the adiposity and body weight gain. This microbiota can also reduce the AMP-activated protein kinase (AMPK) activation and fatty acid oxidation (Kobyliak et al., 2016). Although the literature reports increasing in weight gain and adiposity, we did not observe significant change in anthropometric parameters throughout the study. Briskey et al. (2022) and Cardile et al. (2015) showed significant reduction of body mass, BMI, hip and waist circumferences, fat mass, and fat distribution in overweight adults however these studies used Moro orange extract daily for 3 or 6 months. As in these studies, we also observed significant changes in the cardiometabolic markers in response to MOJ intake, especially in women with insulin resistance. This can also be easily explained by the ability of the bioactive compounds in the MOJ to regulate metabolism (Lima and Barbosa, 2021).

Recent studies have identified novel polymorphisms (Fraga et al., 2022) and microRNAs (Dorna et al., 2021; Quintanilha et al., 2022; Capetini et al., 2023), which regulate cellular processes and mechanisms that are associated with the benefits of orange juice intake. This is one of the few studies that has evaluated the effects of MOJ on the gut microbiota composition and is the first study to report the correlation between the gut microbiota composition and the cardiometabolic biomarkers. Our results showed that specific OTUs play a significant role in the cardiometabolic disease process.

The main limitation of this study was small sample size (12 volunteers). Although we observed clear correlation between several biomarkers and bacterial OTUs, there were large variations in the gut microbiota composition between individuals. This may have affected the significance of several responses and limited the identification of some effects of MOJ intake. Therefore, future studies with larger sample sizes are required to confirm our findings, improve the gut microbiota results, and further explore the benefits associated with MOJ intake.

## 5. Conclusion

In conclusion, MOJ intake had effects on specific OTUs, although significant changes in the gut microbiota composition of overweight women were not observed. MOJ intake increased the production of SCFAs, especially propanoic and isobutyric acids, and improved cardiometabolic biomarkers such as DBP and plasma VCAM-1. The gut microbiota was associated with the levels of SCFAs and cardiometabolic biomarkers. Positive and negative correlations were observed between specific gut microbiota OTUs belonging to the *Bacteroides* and *Prevotella 9* genera, and the cardiometabolic biomarkers at 2-weeks and 4-weeks of MOJ intake. This shows that the gut microbiota played a significant role in flavanone metabolism and influenced the biological processes responsible for the health benefits of MOJ intake.

Finally, the improvements in the cardiometabolic biomarkers were significantly higher in insulin-resistant women including a significant improvement in insulin resistance. The MOJ intake time was important since some changes were significant after 2 weeks of MOJ intake, but others became significant only after 4 weeks. However, future studies are required to confirm our findings and investigate further the effects of MOJ on the gut microbiota and the role of gut microbiota in the health benefits of orange juice.

## Data availability statement

The datasets presented in this study can be found in online repositories. The names of the repository/repositories and accession number(s) can be found at: https://www.ncbi.nlm.nih.gov/, PRJNA508648.

## Ethics statement

The studies involving human participants were reviewed and approved by School of Public Health, University of São Paulo (CAAE 69382217.9.0000.5421), and was registered at the Brazilian Registry of Clinical Trials (UTN: U1111-1241-4665). The patients/participants provided their written informed consent to participate in this study.

## Author contributions

FL, NH, MR, CT, and CH: conceptualization. TC, BQ, and VC: investigation and formal analysis. TC and ECT: data curation. TC, BQ, VC, and RC: methodology. FL, NH, and MR: resources. FL: supervision. TC and ET: writing–original draft. TC, ET, NH, FL, MR, and RC: writing–review and editing. All the authors have read and agreed to the published version of the manuscript.

## Funding

This research was funded by the National Council for Scientific and Technological Development (CNPq) (Grant No. 434713/2018–0) and the São Paulo Research Foundation (FAPESP) (Grant No. 2013/07914–8). The authors are grateful for the scholarships provided by the FAPESP (Grant No. 2020/06467–1) and the CNPq (Grant No. 153624/2018–3).

## Conflict of interest

The authors declare that the research was conducted in the absence of any commercial or financial relationships that could be construed as a potential conflict of interest.

## Publisher’s note

All claims expressed in this article are solely those of the authors and do not necessarily represent those of their affiliated organizations, or those of the publisher, the editors and the reviewers. Any product that may be evaluated in this article, or claim that may be made by its manufacturer, is not guaranteed or endorsed by the publisher.
