# Supplementary material for: Blood orange juice intake changes specific bacteria of gut microbiota associated with cardiometabolic biomarkers
Source: Front Microbiol. 2023 Jul 4;14:1199383. doi: 10.3389/fmicb.2023.1199383 (PMC10352659; doi:10.3389/fmicb.2023.1199383)
Supplement: Supplementary file 1 [file Presentation_1.PDF]

Supplementary Material

Blood Orange Juice Intake Changes Specific Bacteria of Gut Microbiota Associated with Cardiometabolic Biomarkers

Telma A. Faraldo Corrêa[1,2†], Eric de Castro Tobaruela[1,2†*], Vinicius Cooper Capetini[2,3], Bruna Jardim Quintanilha[2,3], Ramon Vitor Cortez[4], Carla R. Taddei[4], Neuza Mariko Aymoto Hassimotto[1,2], Christian Hoffmann[1,2], Marcelo Macedo Rogero[2,3], Franco Maria Lajolo[1,2]

*** Correspondence:**
Eric de Castro Tobaruela
erictobaruela@usp.br

† These authors have contributed equally to this work and share first authorship

Material and Methods

Chemicals

Chemicals were obtained from Sigma-Aldrich (MO, USA) and Merck (Darmstadt, Germany) as elaborated below. Authentic standards of hesperidin, narirutin, didymin, and cyanidin-3-*O*-glucoside were obtained from Extrasynthese (Genay, France).

Quality parameters

The pH and the total soluble sugar (TSS) of Moro orange juice were determined according to the Association of Official Analytical Chemists (AOAC) (Horwitz and Latimer, 2006) and measured using a digital refractometer KRÜSS DR201-95 (Krüss-Optronic GmbH, Hamburg, Germany) and a Metrohm pH meter (827 pH Lab, Metrohm, Herisau, Switzerland), respectively. Three measures were taken at three bottles of orange juice and average values were calculated.

Quantification of soluble sugar, organic acids, and chemical composition

The soluble sugars were analyzed by high performance liquid chromatography (HPLC) coupled to a pulse amperometric detector according to Shiga et al. (2011). The organic acid contents were analyzed by HPLC in a HP1100 system (Hewlett-Packard Company, CA, USA) coupled with a diode-array detector, equipped with a μBondpack C18 (300 mm × 3.6 mm i.d., Waters, MA, USA) and elution (flow rate of 0.5 $mL \cdot min^{-1}$) was carried out in isocratic conditions with 0.1% H_3PO_4, monitored at 210 nm. The content of total dietary fiber and fractions were measured according to the method described by Association of Official Analytical Chemists (AOAC 991.43) (AOAC, 1995).

Analysis of total phenolic content

The total phenolic content of Moro orange juice was determined using the Folin−Ciocalteu colorimetric method described by Singleton and Rossi (1965) with some modifications. A previously shaken orange juice sample (2 mL) was added to 10 mL of methanol/water (80:20, v/v). The sample was vortexed for 1 min and then placed in an ultrasonic bath (15 min) according to the procedure described by Stella et al. (2011). Afterward, the samples were centrifuged at 10000 × *g* during 15 min at 20 °C, and supernatants were filtered by a Whatman filter and analyzed. The absorbance was measured spectrophotometrically at 763 nm. Measurements were recorded on an UV−vis spectrophotometer Helios Epsilon (Thermo Scientific, WI, USA). The results were expressed as milligrams of gallic acid per 100 mL of blood orange juice.

Quantification of flavonoids

Moro orange juice (10 mL) was centrifuged at 10,000 × *g* for 15 min at 4 °C. The supernatant was eluted in a column of 1 g of polyamide (CC 6, Macherey–Nagel), previously preconditioned by passing methanol followed by deionized water. The phenolic compounds were eluted with methanol acidified with 2.5% acetic acid. The eluates were completely dried by rotary evaporation (Rotavapor, RE 120, Flawil, Switzerland) under a vacuum at 40 °C, resuspended with methanol acidified with 5% acetic acid, and filtered through a 0.45 μm PVDF Millex filter (Millipore, MA, USA) before HPLC

analysis. The pellet was added to 20 mL of dimethyl sulfoxide, homogenized overnight at room temperature, centrifuged, and filtered through a 0.45 μm PVDF filter.

Samples were analyzed by HPLC on Agilent 2100 equipment coupled to a diode array detector (DAD) using a Prodigy 5 μm ODS3 column (250 × 4.60 mm) (Phenomenex, Cheshire, UK) with a flow rate of 1 $mL \cdot min^{-1}$ at 25 °C. Elution was carried out with a solvent gradient constituted of 0.5% formic acid in water (A) and 0.5% formic acid in acetonitrile (B). The solvent concentration gradient applied was 8% B at the beginning, 10% for 5 min, 17% for 10 min, 25% for 15 min, 50% for 25 min, 90% for 30 min, 50% for 32, and 8% for 35 min. The eluates were monitored at 280 and 525 nm (Brasili et al., 2017). Quantification was performed using a calibration curve of cyanidin-3-*O*-glucoside (at 525 nm) and narirutin, hesperidin, and didymin at 280 nm. Cyanidin-3-*O*-(6″-malonyl)glucoside was quantified as cyanidin-3-*O*-glucoside equivalent.

Peak identification was carried out by Prominence liquid chromatography (Shimadzu, Japan) coupled to an ion trap mass spectrometer (Esquire HCT model, Bruker Daltonics, MA, USA). The separation conditions were the same as those used for HPLC/DAD, and the flow rate was changed to 0.2 $mL \cdot min^{-1}$ to allow the eluate to pass through the mass spectrometer. The ESI was maintained in positive and negative modes for anthocyanins and other flavonoid classes, respectively. The mass operating conditions were programed to perform a full scan (*m/z* 100–1000), with a collision energy of 3000–3500 V, and a capillary temperature of 275 °C. Peak identification was carried out by the combined information provided by mass spectra, retention time, and literature data (Hillebrand et al., 2004). The identity of hesperidin, narirutin, didymin, and cyanidin-3-*O*-glucoside was confirmed by co-elution with authentic standards.

References

Association of Official Analytical Chemists (AOAC). Official Methods of Analysis, 16th ed.; AOAC International: Arlington, VA, USA, 1995.

Brasili, E.; Chaves, D. F.; Xavier, A. A.; Mercadante, A. Z.; Hassimotto, N. M. A.; Lajolo, F. M. Effect of Pasteurization on Flavonoids and Carotenoids in Citrus sinensis (L.) Osbeck cv. 'Cara Cara' and 'Bahia' Juices. *J. Agric. Food Chem.* 2017, *65(7)*, 1371–1377.

Hillebrand, S.; Schwarz, M.; Winterhalter, P. Characterization of Anthocyanins and Pyranoanthocyanins from Blood Orange [Citrus sinensis (L.) Osbeck] Juice. *J. Agric. Food Chem.* 2004, *52(24)*, 7331–7338.

Horwitz, W.; Latimer, G. W. J. Official methods of analysis of AOAC International. 18th. ed. Gaithersburg: AOAC International; 2006. 2590 p.

Shiga, T.; Soares, C. A.; Nascimento, J. R. O.; Purgatto, E.; Lajolo, F. M.; Cordenunsi, B. R. Ripening-associated changes in the amounts of starch and non-starch polysaccharides and their contributions to fruit softening in three banana cultivars. *J. Sci. Food Agric.* 2011, *91(8)*, 1511–1516.

Singleton, V. L.; Rossi, J. A. Colorimetry of total phenolics with phosphomolybdic-phosphotungstic acid reagents. *Am. J. Enol. Vitic.* 1965, *16*, 144−158.

Stella, S. P.; Ferrarezi, A. C.; Santos, K. O.; Monteiro, M. Antioxidant activity of commercial ready-to-drink orange juice and nectar. *J. Food Sci.* 2011, 76, 392−397.

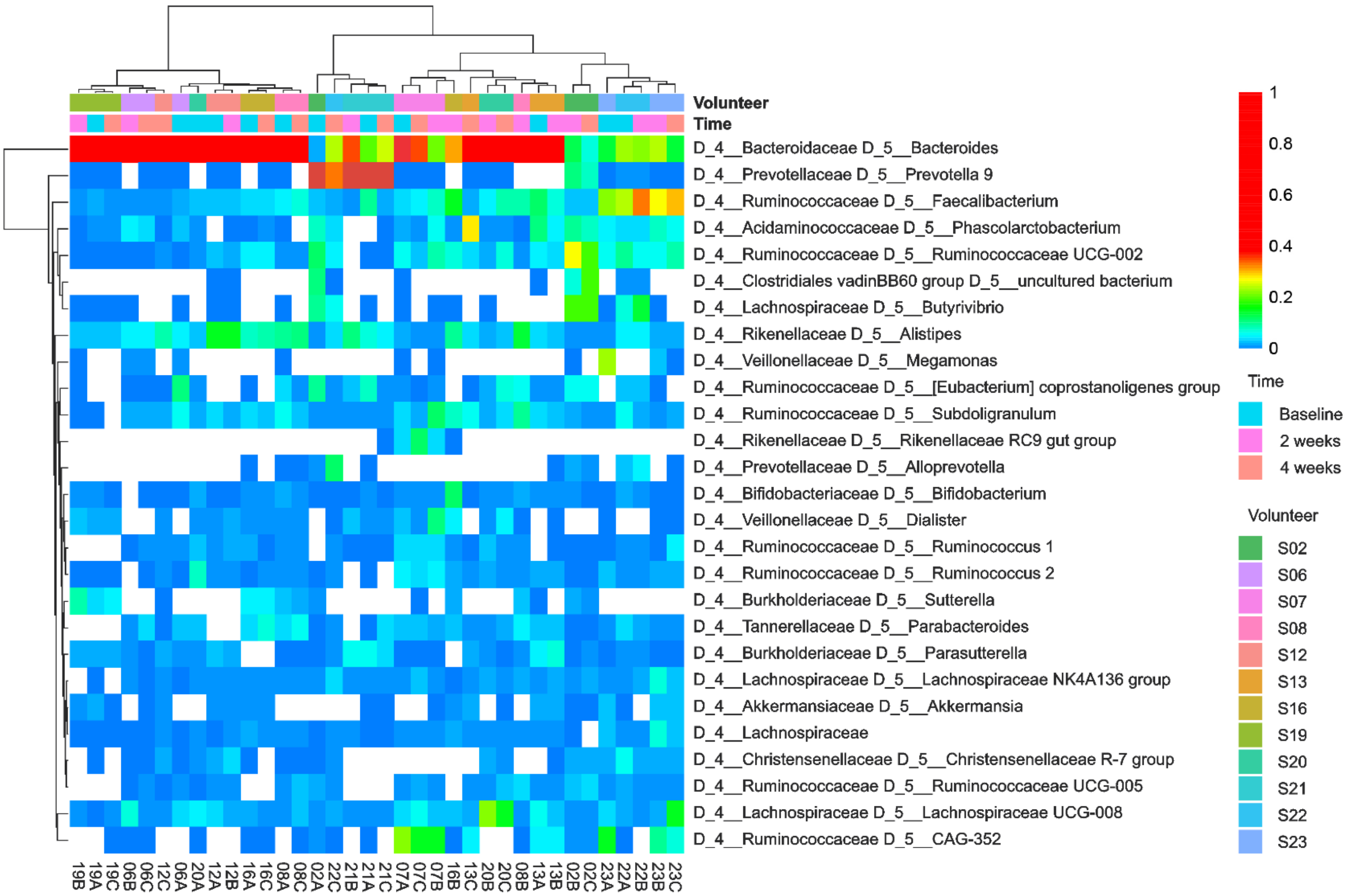

Supplementary Figure 1. Heatmap and hierarchical clustering shows the gut microbiota composition of the study volunteers at baseline and at 2-weeks and 4-weeks time points during the Moro orange juice intake. Each row represents an operational taxonomic unit (OTU), and each column represents a sample as indicated. The color legend at the top of the graph indicates the time point and the volunteer identification.

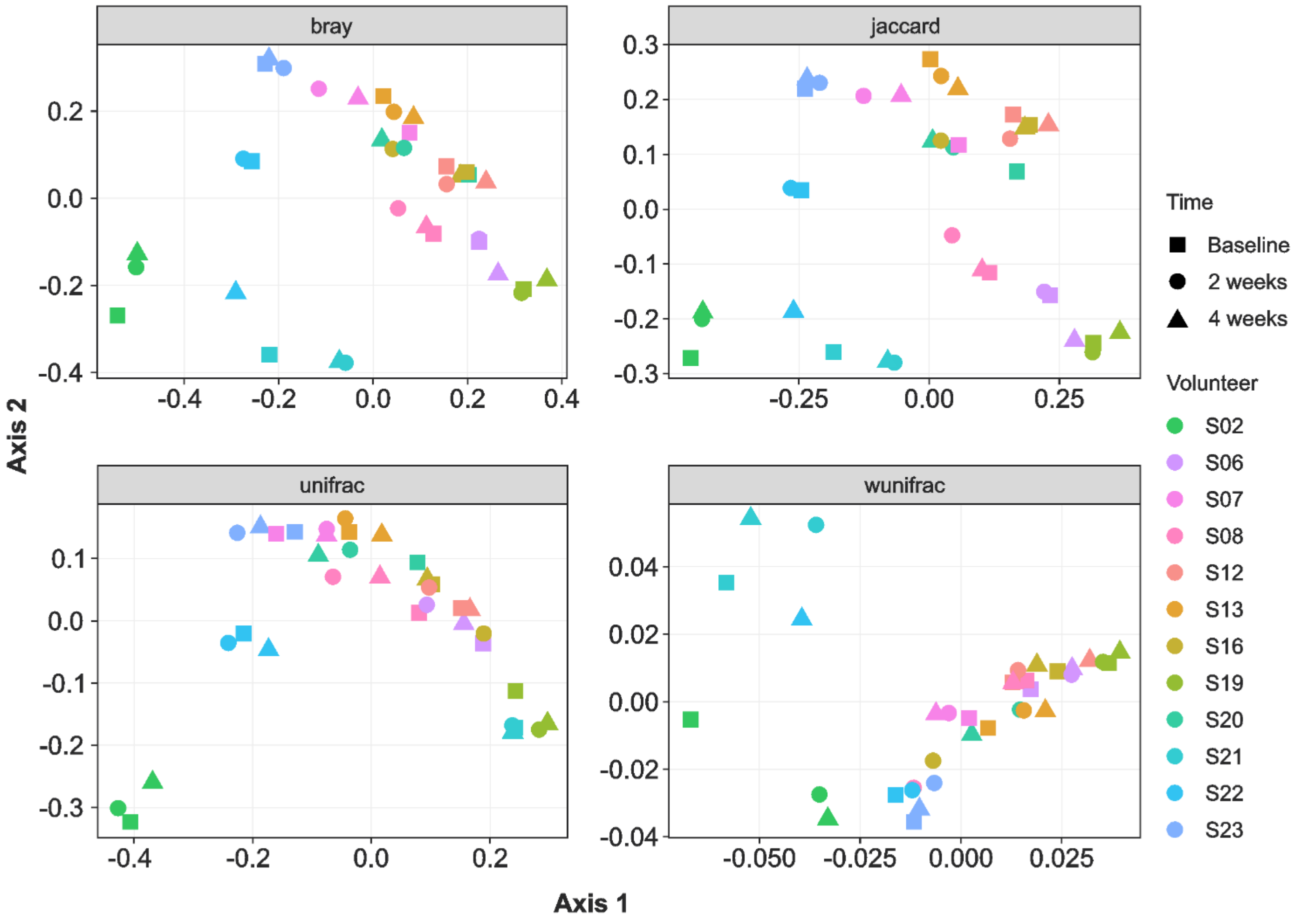

Supplementary Figure 2. A summary of the gut microbiome analysis of the samples from 12 volunteers at baseline and at 2-weeks and 4-weeks time points during the Moro orange juice intake is displayed using principal coordinate analysis (PCoA) plots. Gut microbiota beta-diversity is presented based on the 4 common indices (Bray-Curtis, Jaccard index, unifrac, and wunifrac). The shape and color of the marker indicates the time point and the volunteer identification, respectively.

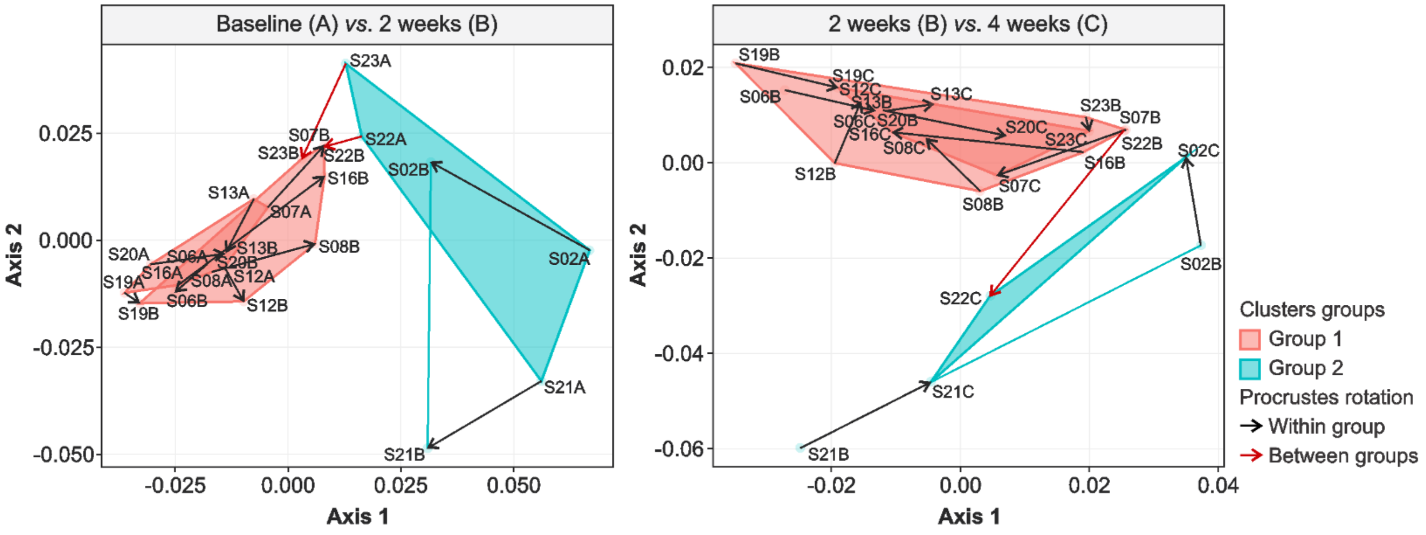

Supplementary Figure 3. Procrustes analysis of the gut microbiome using weighted UniFrac distances and principal coordinate analysis (PCoA) ordination. The distribution of the 12 volunteers at baseline and at 2-weeks and 4-weeks time points during the Moro orange juice intake is displayed using PCoA plots. The color of lines and arrows indicates the cluster group and the volunteer rotations within or between groups, respectively.

Supplementary Table 1. Quality parameters and chemical composition of the pasteurized Moro orange juice

	Moro orange juice
Quality Parameters	
pH	3.48 ± 0.00
TSS (°Brix)	10.00 ± 0.00
Soluble sugars (mg·500 mL^{-1})	
Sucrose	14.00 ± 0.72
Fructose	9.50 ± 0.43
Glucose	8.45 ± 0.52
Total	31.95
Organic acids (mg·500 mL^{-1})	
Citric acid	5809.70 ± 234.65
Malic acid	1369.85 ± 53.47
Ascorbic acid	160.00 ± 7.51
Tartaric acid	131.25 ± 2.75
Total	7470.80
Total phenolic compounds (mg·500 mL^{-1})	303.75 ± 0.03
Dietary fiber (g·500 mL^{-1})	
Soluble dietary fiber	0.40 ± 0.03
Insoluble dietary fiber	1.30 ± 0.06
Total dietary fiber	1.70

TSS: Total soluble solids. Results are expressed as the mean ± standard error (n = 3 bottles).

Supplementary Table 2. Identity and mass spectrometric properties of flavanones and anthocyanins of pasteurized Moro orange juice

Flavanone	RT (min)	$[M–H]^-$ (*m/z*)	MS/MS fragment ions (*m/z*)
Naringenin-7-*O*-rutinoside (Narirutin)	16.6	579.39	271.23
Hesperitin-7-*O*-rutinoside (Hesperidin)	19.3	609.43	301.24
Isosakuranetin-7-*O*-rutinoside (Didymin)	24.6	593.96	285.24
**Anthocyanin**	**RT (min)**	**$[M]^+$ (*m/z*)**	**MS/MS fragment ions (*m/z*)**
Cyn-3-*O*-glu	13.3	449.19	287.07
Cyn-3-*O*-(malonyl)glu*	18.1	535.17	449.19/287.07

Cyn-3-*O*-glu: cyanidin-3-*O*-glucoside. Cyn-3-*O*-(malonyl)glu: cyanidin-3-*O*-(6″-malonyl)glucoside. RT: Retention time. *Structure identification based on Mullen et al., (2003) and Hillebrand et al. (2004).

References

Mullen, W.; Yokota, T.; Lean, M. E. J.; Crozier, A. Analysis of ellagitannins and conjugates of ellagic acid and quercetin in raspberry fruits by LC–MSn. *Phytochemistry.* 2003, *64*, 617–624.

Hillebrand, S.; Schwarz, M.; Winterhalter, P. Characterization of Anthocyanins and Pyranoanthocyanins from Blood Orange [*Citrus sinensis* (L.) Osbeck] Juice. *J. Agric. Food Chem.* 2004, *52*, 7331–7338.

Supplementary Table 3. Flavonoid content (mg/500 mL) in the pasteurized Moro orange juice

Flavonoid	Supernatant	Pellet	Total
Flavanone			
Narirutin	12.40 ± 1.40	8.55 ± 4.80	20.95
Hesperidin	29.45 ± 3.10	152.80 ± 67.90	182.25
Didymin	3.40 ± 0.80	6.10 ± 3.45	9.50
Anthocyanin			
Cyn-3-*O*-glu	61.15 ± 8.80	N.D.	61.15
Cyn-3-*O*-(malonyl)glu	14.45 ± 5.70	N.D.	14.45
**Total Flavanones**	**45.25**	**167.45**	**212.70**
**Total Anthocyanins**	**75.60**	**N.D.**	**75.60**
**Total Flavonoids**	**120.85**	**167.45**	**288.30**

Cyn-3-*O*-glu: cyanidin-3-*O*-glucoside. Cyn-3-*O*-(malonyl)glu: cyanidin-3-*O*-(6″-malonyl)glucoside (quantified as cyanidin-3-*O*-glucoside equivalent). N.D.: Not detected. Results are expressed as the mean ± standard error (n = 3 bottles).

Supplementary Table 4. Relative abundance of significant gut microbiota phyla in overweight women at baseline and at 2-week and 4-week time points during the Moro orange juice intake

	Baseline	2 weeks	4 weeks	*p*-value
Bacteroidetes	54.07 ± 6.09	47.61 ± 6.72	59.01 ± 7.64	0.513
Firmicutes	38.85 ± 5.85	44.80 ± 7.04	34.93 ± 7.02	0.532
Proteobacteria	3.36 ± 0.51	4.37 ± 1.20	3.42 ± 0.52	1.000
Actinobacteria	**0.70 ± 0.22**^{a}	**1.26 ± 0.83**^{ab}	**0.28 ± 0.09**^{b}	**0.043**
Verrucomicrobia	0.82 ± 0.23	0.49 ± 0.15	0.71 ± 0.25	0.627
Tenericutes	0.46 ± 0.28	0.53 ± 0.27	0.42 ± 0.30	0.748
Unknown	0.27 ± 0.20	0.32 ± 0.22	0.19 ± 0.09	0.993
Others	1.48 ± 1.14	0.63 ± 0.37	1.05 ± 0.91	0.986

Results are expressed as the mean ± standard error ($n = 12$). *P*-values were calculated using the Kruskal-Wallis Test. Values in bold and different superscript letters indicate statistical significance ($p < 0.05$) among values obtained in the baseline and after 2 and 4 weeks of Moro orange juice intake.

Supplementary Table 5. Relative abundance of significant gut microbiota genera in overweight women at baseline and at 2-week and 4-week time points during the Moro orange juice intake

	Baseline	2 weeks	4 weeks	*p*-value
Bacteroides	38.99 ± 6.99	32.66 ± 6.5	40.51 ± 7.41	0.756
Faecalibacterium	6.01 ± 1.73	5.54 ± 2.17	4.03 ± 1.71	0.416
Alistipes	5.17 ± 1.09	5.10 ± 1.35	4.54 ± 0.92	0.939
Prevotella 9	**6.15 ± 4.31[a]**	**4.30 ± 3.50[b]**	**7.64 ± 4.96[a]**	**0.004**
Ruminococcaceae UCG-002	3.00 ± 0.75	3.16 ± 1.09	3.35 ± 0.90	0.759
Lachnospiraceae UCG-008	2.13 ± 0.49	2.75 ± 1.22	2.68 ± 1.11	0.923
Subdoligranulum	2.05 ± 0.38	2.20 ± 0.72	1.31 ± 0.30	0.480
[Eubacterium] coprostanoligenes group	2.53 ± 0.72	2.17 ± 0.62	1.08 ± 0.41	0.101
Phascolarctobacterium	2.06 ± 0.71	1.82 ± 0.52	3.04 ± 1.74	0.619
Butyrivibrio	**0.82 ± 0.56[b]**	**1.40 ± 0.94[a]**	**0.99 ± 0.71[b]**	**0.001**
Ruminococcus 2	1.58 ± 0.79	1.36 ± 0.54	0.74 ± 0.30	0.130
Parabacteroides	1.64 ± 0.30	1.30 ± 0.29	2.17 ± 0.49	0.297
CAG-352	**2.40 ± 1.38[a]**	**1.26 ± 0.89[b]**	**1.72 ± 0.98[a]**	**0.010**
Dialister	0.62 ± 0.25	1.08 ± 0.58	0.75 ± 0.31	0.198
Christensenellaceae R-7 group	1.07 ± 0.57	1.07 ± 0.39	0.77 ± 0.31	0.310
Lachnospiraceae NK4A136 group	0.80 ± 0.22	1.07 ± 0.30	1.27 ± 0.47	0.473
Sutterella	0.83 ± 0.42	0.99 ± 0.59	0.85 ± 0.43	0.069
Parasutterella	0.90 ± 0.40	0.96 ± 0.44	0.58 ± 0.28	0.086
Ruminococcaceae UCG-005	0.67 ± 0.23	0.92 ± 0.34	0.98 ± 0.30	0.604
Ruminococcus 1	0.98 ± 0.27	0.82 ± 0.27	0.93 ± 0.39	0.352
Desulfovibrio	0.63 ± 0.18	0.69 ± 0.37	0.47 ± 0.13	0.263
Ruminococcaceae UCG-010	**0.52 ± 0.30[b]**	**0.55 ± 0.26[b]**	**0.72 ± 0.48[a]**	**0.042**
Roseburia	0.57 ± 0.11	0.55 ± 0.19	0.45 ± 0.18	0.110
[Eubacterium] eligens group	**0.16 ± 0.08[b]**	**0.54 ± 0.25[a]**	**0.16 ± 0.05[b]**	**<0.001**
Megasphaera	**0.10 ± 0.07[b]**	**0.39 ± 0.26[ab]**	**0.64 ± 0.43[a]**	**0.036**
Bifidobacterium	**0.52 ± 0.19[a]**	**0.35 ± 0.09[ab]**	**0.17 ± 0.08[b]**	**0.007**
Lachnoclostridium	**0.65 ± 0.17[a]**	**0.33 ± 0.05[b]**	**0.41 ± 0.10[ab]**	**<0.001**
Alloprevotella	**0.15 ± 0.11[b]**	**0.33 ± 0.23[ab]**	**0.92 ± 0.90[a]**	**0.020**
Escherichia-Shigella	**0.13 ± 0.04[b]**	**0.32 ± 0.12[ab]**	**0.64 ± 0.27[a]**	**0.001**
Rikenellaceae RC9 gut group	0.23 ± 0.23	0.29 ± 0.25	1.17 ± 1.16	0.113
Akkermansia	**0.68 ± 0.23[a]**	**0.27 ± 0.11[b]**	**0.67 ± 0.26[a]**	**0.037**
Megamonas	**1.36 ± 1.32[a]**	**0.10 ± 0.07[b]**	**0.01 ± 0.01[b]**	**0.007**
Uncultured	3.39 ± 1.23	2.62 ± 1.08	3.36 ± 1.59	0.793
Unknown	2.24 ± 0.52	1.86 ± 0.53	1.74 ± 0.43	0.617
Others	8.27 ± 1.10	13.31 ± 4.19	8.53 ± 1.23	0.662

Results are expressed as the mean ± standard error ($n = 12$). *P*-values were calculated using the Kruskal-Wallis Test. Values in bold and different superscript letters indicate statistical significance ($p < 0.05$) among values obtained in the baseline and after 2 and 4 weeks of Moro orange juice intake.

Supplementary Table 6. Gut microbiota diversity analysis. Number of observed species and alpha-diversity measures

Volunteer	Time	Observed species	Alpha-diversity			
			Shannon	Simpson	S.chao1	Se.chao1
S02	Baseline	2223	7.27	0.97	3052.01	76.03
	2 weeks	2752	8.17	0.99	4005.01	99.94
	4 weeks	1663	7.39	0.98	2369.02	73.96
S06	Baseline	504	5.10	0.92	694.56	39.76
	2 weeks	748	5.43	0.93	1038.82	48.40
	4 weeks	529	4.44	0.83	765.39	47.08
S07	Baseline	1327	6.33	0.95	2072.36	86.14
	2 weeks	974	6.10	0.96	1411.04	61.52
	4 weeks	1004	5.97	0.95	1428.01	59.01
S08	Baseline	626	5.74	0.95	801.38	34.13
	2 weeks	1117	6.62	0.98	1494.00	51.31
	4 weeks	815	5.89	0.95	1056.57	38.90
S12	Baseline	561	5.54	0.93	740.25	36.18
	2 weeks	768	5.67	0.94	1133.73	58.34
	4 weeks	578	4.90	0.90	767.00	36.29
S13	Baseline	858	5.78	0.95	1236.51	56.94
	2 weeks	895	5.66	0.93	1288.37	58.21
	4 weeks	630	4.95	0.90	927.73	52.98
S16	Baseline	677	5.13	0.91	937.37	44.56
	2 weeks	387	5.65	0.96	496.31	28.50
	4 weeks	701	5.26	0.92	977.12	45.64
S19	Baseline	474	3.98	0.77	680.90	41.44
	2 weeks	356	3.94	0.82	478.10	30.93
	4 weeks	312	3.54	0.77	418.89	30.91
S20	Baseline	701	5.00	0.88	885.24	32.12
	2 weeks	850	5.43	0.91	1122.00	42.19
	4 weeks	869	5.85	0.96	1421.02	79.93
S21	Baseline	462	4.77	0.90	597.01	30.52
	2 weeks	474	4.78	0.89	738.05	55.48
	4 weeks	422	4.37	0.84	600.36	41.18
S22	Baseline	1260	6.85	0.98	1770.25	62.69
	2 weeks	1287	6.48	0.96	1781.13	60.48
	4 weeks	1061	5.93	0.94	1409.40	47.68
S23	Baseline	867	5.89	0.95	1219.35	51.51
	2 weeks	1358	6.69	0.97	1954.53	69.25
	4 weeks	1236	6.39	0.97	1897.78	80.72

Supplementary Table 7. Energy, macronutrient, and micronutrient intake calculated through the information obtained with the 24 h dietary recall conducted at baseline and at 2-week and 4-week time points during the Moro orange juice intake

	Baseline	2 weeks	4 weeks	*p*-value
Energy (kcal·100 g^{-1})	2176.44 ± 133.80	2375.21 ± 110.43	2344.90 ± 140.56	0.076
Carbohydrates (g·100 g^{-1})	308.39 ± 15.53	310.81 ± 24.44	280.99 ± 16.71	0.338
Proteins (g·100 g^{-1})	76.43 ± 5.86	86.70 ± 8.55	80.54 ± 7.70	0.558
Lipids (g·100 g^{-1})	76.12 ± 8.05	86.67 ± 7.07	93.40 ± 10.94	0.779
Cholesterol (mg·100 g^{-1})	293.83 ± 45.18	304.91 ± 52.20	311.91 ± 47.74	0.779
Dietary fiber (g·100 g^{-1})	**20.26 ± 2.70[a]**	**19.98 ± 2.68[a]**	**13.51 ± 1.52[b]**	**0.039**
Calcium (mg·100 g^{-1})	478.99 ± 82.54	496.74 ± 75.16	598.00 ± 70.06	0.779
Magnesium (mg·100 g^{-1})	182.54 ± 21.39	201.25 ± 14.30	189.35 ± 18.39	0.779
Manganese (mg·100 g^{-1})	2.03 ± 0.23	1.86 ± 0.41	1.46 ± 0.21	0.368
Phosphor (mg·100 g^{-1})	958.93 ± 76.79	1039.90 ± 114.35	947.81 ± 103.34	0.717
Iron (mg.100 g^{-1})	8.88 ± 1.73	8.95 ± 1.06	6.77 ± 0.66	0.076
Sodium (mg·100 g^{-1})	1667.90 ± 122.92	2186.41 ± 235.85	2038.64 ± 295.22	0.205
Potassium (mg·100 g^{-1})	**2261.17 ± 230.09[b]**	**2823.73 ± 113.64[a]**	**2683.95 ± 195.14[ab]**	**0.039**
Cupper (mg·100 g^{-1})	2.54 ± 1.32	1.08 ± 0.26	0.64 ± 0.06	0.472
Zinc (mg·100 g^{-1})	7.89 ± 1.14	9.56 ± 2.07	9.08 ± 1.24	0.920
Vitamin C (mg·100 g^{-1})	**66.55 ± 27.95[b]**	**297.24 ± 23.33[a]**	**278.13 ± 18.55[a]**	**<0.001**
Vitamin A (RE) (µg·100 g^{-1})	262.35 ± 76.18	267.51 ± 69.33	123.31 ± 47.00	0.144
Vitamin A (RAE) (µg·100 g^{-1})	133.73 ± 38.76	138.11 ± 34.16	63.21 ± 23.72	0.127
Retinol (µg·100 g^{-1})	320.13 ± 142.96	183.5 ± 31.79	232.97 ± 73.38	0.558
Thiamine (mg·100 g^{-1})	0.92 ± 0.28	0.93 ± 0.09	1.12 ± 0.27	0.205
Riboflavin (mg·100 g^{-1})	1.02 ± 0.22	0.93 ± 0.12	1.08 ± 0.18	0.517
Pyridoxine (mg·100 g^{-1})	0.95 ± 0.28	0.95 ± 0.09	1.07 ± 0.11	0.338
Niacin (mg·100 g^{-1})	14.47 ± 3.07	12.99 ± 2.77	13.54 ± 2.91	0.779

Results are expressed as the mean ± standard error ($n = 12$). The *p*-values were calculated using the Friedman test. Values in bold and different superscript letters indicate statistical significance ($p < 0.05$) among values obtained in the baseline and after 2 and 4 weeks of Moro orange juice intake.
